# The association between self-reported mobile phone usage with blood pressure and heart rate: evidence from a cross-sectional study

**DOI:** 10.1186/s12889-022-14458-1

**Published:** 2022-11-07

**Authors:** Fatemeh Amiri, Mehdi Moradinazar, Jalal Moludi, Yahya Pasdar, Farid Najafi, Ebrahim Shakiba, Behrooz Hamzeh, Amir Saber

**Affiliations:** 1 Radiology and Nuclear Medicine Department, School of Paramedical, Kermanshah University of Medical Sciences, Kermanshah, Iran; 2 Behavioral Disease Research Center, Kermanshah University of Medical Sciences, Kermanshah, Iran; 3 Department of Nutritional Sciences, School of Nutritional Sciences and Food Technology, Kermanshah University of Medical Sciences, Kermanshah, Iran; 4 Research Center for Environmental Determinants of Health, School of Public Health, Kermanshah University of Medical Sciences, Kermanshah, Iran

**Keywords:** Mobile phone, Hypertension, Systolic blood pressure, Diastolic blood pressure

## Abstract

**Background:**

With the advancement of technology, the rate of access and use of mobile phones in different communities has increased significantly. Mobile phones emit electromagnetic waves and therefore excessive use of them may have harmful effects on physical and mental health and especially on the cardiovascular system. This study aimed to investigate the association between self-reported mobile phone use duration and blood pressure and heart rate (HR) using data from Ravansar non-communicable diseases (RaNCD) cohort study.

**Methods:**

The present cross-sectional study was performed using the data of 8905 out of 10,065 participants in the RaNCD study in Iran. According to the mean self-reported duration of mobile phone usage (min/day) over the previous 12 months, all users were divided into four groups. The first and fourth groups had the least and most time using mobile phones respectively. The relationship between blood pressure and the duration of mobile phone use was determined using univariate and multiple linear regression.

**Results:**

Of 8905 participants aged 35–65 years, 1515 (17.0%) of them didn't use mobile phones. The minimum, maximum, and mean duration of self-reported mobile phone use between users were 3.4, 50.4, and 19.5 min/day, respectively. A decrease in women's systolic and diastolic blood pressure (SBP and DBP) and HR was observed by increasing the duration of mobile phone use. With adjustment for effective confounding factors, there was a significant negative association between SBP [-2.52 (-4.11, -0.94)], DBP [-1.86 (-2.83, -0.89)], and duration of mobile use.

**Conclusion:**

In this study, a significant decreasing trend was found between SBP, DBP, and HR and higher mobile phone usage in women. Based on regression analysis, SBP, DBP, and duration of mobile phone use were associated negatively in those who used their phones for at least 8 h.

## Background

The use of cell phones has dramatically increased over the past two decades, with seven billion mobile subscribers registered worldwide, including 5.4 billion in developing countries [1, 2]. The number of mobile phone users is expected to exceed five billion by 2019, and this number reached 266 million in the US in 2017 and exceeded 48 million in Iran [3]. Although cell phone is considered as a valuable and useful Information Communication Technology tool (ICT), it can have adverse health consequences [1, 4–6]. According to numerous studies, the short-term effects of mobile phone use include changes in sleep patterns, blood pressure, and HR. However, the potential relationship of mobile phone use with cardiovascular disease (CVD) and hypertension has not yet been elucidated due to the reported contradictory results [7–9].

Hypertension is a leading preventable cause of CVD mortality and disease burden worldwide and is known as an important risk factor for ischemic heart disease, stroke, chronic kidney disease, and dementia [10–12]. Based on recent estimation 1.4 billion people worldwide have high blood pressure and 7.7–10.4 million annual deaths are attributable to high blood pressure [13–15]. In addition, different epidemiological studies have shown that the high prevalence of hypertension has shifted from developed countries to moderate and low-income countries in recent decades, and this rate has increased in East, South, and Southeast Asia, Oceania, and sub-Saharan Africa [13, 16, 17]. Contradictory results have been reported for the relationship between blood pressure and mobile phone use; for instance, the risk of developing hypertension was found to increase with the duration of mobile phone use in 2018. This risk was also found to increase six-fold in individuals who had been using mobile phones for at least eight years and four-fold in those using over 60 min/day [5]. In contrast, using mobile phones was found to reduce blood pressure by decreasing stress and thereby developing social relationships [7]. Stalin et al. also reported negative relationships between blood pressure and using mobile phones in India [18]; On the other hand, many studies reported no relationships between mobile phone use and blood pressure [19, 20].

There is no study has been done yet inside the country on the relationship between mobile phone usage and CVD and hypertension and this is the first study conducted in Iran to investigate the relationship. Given the moderate prevalence of hypertension in Iran (32%) and increasing trend of mobile phone use, as a common communication technology, and also the obscure relationship between mobile phone use and blood pressure, the present study was conducted to investigate the relationship between self-reported mobile phone use and SBP, DBP, and HR using large data from Ravansar Non-Communicable Disease (RaNCD) cohort study.

## Methods

### Study design

This cross-sectional study was performed using data collected from the recruitment stage of the RaNCD cohort study. The RaNCD cohort study is a population-based prospective study that has been conducted on 10,065 permanent residents consisting of men and women aged 35–65 years in Ravansar (one of the cities of Kermanshah province in Iran) to investigate the results of chronic diseases and death since 2014 [21, 22]. This study is one of the parts of Iran’s mega-cohort study called the PERSIAN cohort (Prospective Epidemiological Research Studies in Iran) [23].

Ravansar is a region with urban and rural areas located in western Iran in the Kermanshah province near the Iraq border which has about 50,000 populations of Kurdish ethnicity. Out of approximately 15,000 eligible candidates among Ravansar residents, 10,000 were included in the present study. Further information can be obtained from the cohort profile [22, 23]. This study was approved by the ethics committee of Kermanshah University of medical sciences with code No. IR. KUMS. REC.1394.318 and conducted in accordance with the Declaration of Helsinki.

### Data collection and quality control

For data collection, a trained expert informed the eligible candidates about the study design and objectives and invited those willing to participate in the cohort study to the study center. All of the selected subjects underwent comprehensive physical examinations and participated in a comprehensive face-to-face medical interview in a private setting according to the cohort protocol. Written consent has been obtained from all participants. The collected data were investigated by the center supervisor and ultimately recorded after ensuring their accuracy on the same day. In case of any problem, follow-up was performed and the subjects were asked to present in the cohort center and complete the questionnaire again. In this study, the online version of the standard questionnaires was used to collect information, in accordance with the PERSIAN cohort protocol. However, the questionnaires used in this study were revised after performing a pre-pilot phase to improve their validity and reliability [22, 23]. The socioeconomic status of participants was evaluated by a comprehensive questionnaire about employment status, education, employment history, marital status, number and type of marriages, spouse’s job, region of residency, number of domestic and international trips, access to a landline, and mobile phones, the internet and the extent of mobile phone use.

The smoking status of participants was evaluated based on the National Health Interview Survey (NHIS). According to this guideline, those who have smoked less than 100 cigarettes in their lifetime were classified as non-smokers, those who have smoked more than 100 cigarettes in the past and still smoking were classified as current smokers, and those who have smoked more than 100 cigarettes in the past but do not smoke now were classified as former smokers [24]. A positive record of alcohol and drug consumption was determined by asking whether they have consumed any kind of alcoholic drink or industrial/domestic drugs in their lifetime. The physical activity level of participants was categorized based on daily metabolic equivalents (METs) and determined cutoff points as low (24–36.5 Mets-hour per week), moderate (36.6–44.9 Mets-hour per week), and severe (≥ 45 Mets-hour per week) [22].

Chronic diseases (Kidney disease, CVD, depression, and psychiatric disorders) were assessed based on self-report and/or medical records [22]. SBP and DBP were measured using a standard manual sphygmomanometer (Riester), after at least 4–5 min of rest in a sitting position, two times from both arms, and the interval between two measurements was 10 min. The mean of the two obtained blood pressure from both arms (left and right) was recorded as the final blood pressure. According to criteria recommended by the eighth report of the Joint National Committee on Prevention, Detection, Evaluation, and Treatment of High BP (JNC8), participants in this study were considered to have high blood pressure when SBP ≥ 140 mm of mercury (mmHg) and/or DBP ≥ 90 mmHg. Besides, all hypertensive patients were excluded from the study [25]. The duration of mobile phone use was measured by asking participants directly about their average duration of use over the past 12 months (containing day, week, and month), including calling or answering. The questions asked to determine the duration of using the mobile phone were as follows: 1) Do you use a mobile phone?, 2) How many years have you been using a mobile phone?, 3) On average, how much time did you spend calling or answering your mobile phone in the last 12 months?, 4) On average, how much time did you spend using your cell phone or tablet to do things other than calling and answering the phone in the last 12 months? (For example, SMS, chatting, playing games, or using the Internet). Your answer should be the number of minutes or hours you use your phone in a typical day, week, or month.

By converting and adjusting mobile phone use time the mean daily duration was determined in minutes and used for analysis. After the exclusion of subjects with missing information about the mobile phone usage (*n* = 35), people with high blood pressure who were taking antihypertensive (*n* = 1063), and people with incomplete data (*n* = 62), a total of 8905 subjects were selected from the 10,065 participants of the cohort study. The participants were classified into four groups (quartile) based on the duration of mobile phone use. The first and fourth groups had the least and most time using mobile phones respectively. Also, the non-users were considered as the reference.

### Statistical analysis

The STATA-14 statistical software package was used for data analysis. The continuous and qualitative variables were reported as mean ± standard deviation, and frequency (%), respectively. Blood pressure and HR were analyzed as quartiles across the duration of mobile phone usage. The trend chi-square test was used to assess the linear trend between them. In order to investigate the association between mobile phone use duration and blood pressure and HR, a univariate and multiple linear regression analysis was performed. Variables with *p* < 0.3 were entered in the multiple model and variables with *p* < 0.05 were kept, and other variables were excluded using a stepwise (Backward) method. All of the linear regression assumptions such as linear relationship, normal distribution, and multicollinearity were checked. In addition, less than one percent of the data was missed and excluded from this study. The significance level of < 0.05 with 95% confidence intervals was considered for statistical analysis.

## Results

In this study, 10,065 participants in the RaNCD cohort study aged 35–65 years were examined in terms of mobile phone use, and a total of 1160 participants (11.5%) were excluded due to incomplete data, taking antihypertensive medicine, and lack of sufficient information about cell phone use. Table 1 shows that, the majority of study participants were in the age range of 35–45 years, and the mean age in males and females was 46.1 ± 8.6 years and 46.2 ± 8.8 years, respectively. Also, 5351 (60.08%) of participants were lived in urban areas, 1106 (12.41%) were current smokers, 602 (6.76%) were drinking alcohol and 2293 (25.74%) of them had BMI ≥ 30 (Table 1). The duration of mobile phone use among participants was in the range of 0–330 min/day with a mean of 19.5 min/day, and a total of 1515 (17.0%) of participants didn't use mobile phones. The total number of participants in each quartile and their mean mobile phone use time were as follow, Q1: *n* = 1949, time = 3.4 min/day; Q2: *n* = 1733, time = 8 min/day; Q3: *n* = 1911, time = 16.2; Q4: *n* = 1797, time = 50.4 min/day (Table 2).

**Table 1:** Baseline characteristics of RaNCD cohort study participants (*n* = 8905)

Variables		Total, n (%)	Male, n (%)	Female, n (%)
**Age group**	35–45	4286 (48.13)	2083 (48.60)	2203 (51.39)
	46–55	2966 (33.30)	1532 (51.65)	1434 (48.34)
	56–65	1653 (18.56)	785 (47.48)	868 (52.51)
**Residential areas**	Urban	5351 (60.08)	2729 (50.99)	2622 (49.00)
	Rural	3554 (39.91)	1671 (47.01)	1883 (52.98)
**Education level**	Illiterate	1957 (21.97)	528 (26.98)	1429 (73.01)
	1–5 years	3435 (38.57)	1262 (36.73)	2173 (63.26)
	6–9 years	1568 (17.60)	1081 (68.94)	487 (31.05)
	10-12 years	1202 (13.49)	931 (77.45)	271 (22.54)
	> 13	743 (8.34)	598 (80.48)	145 (19.51)
**Socioeconomic status**	1st quintile (lowest)	1731 (19.43)	573 (33.10)	1158 (66.89)
	2nd quintile	1734 (19.47)	788 (45.44)	946 (54.55)
	3rd quintile	1797 (20.17)	913 (50.80)	884 (49.19)
	4th quintile	1795 (20.15)	919 (51.19)	876 (48.80)
	5th quintile (highest)	1848 (20.75)	1207 (65.31)	641 (34.68)
**Smoking status**	Non-smokers	7108 (79.82)	2811 (39.54)	4297 (60.45)
	Current smokers	1106 (12.41)	1023 (92.49)	83 (7.50)
	Former smokers	691 (7.75)	573 (82.92)	118 (17.07)
**Alcohol intake**	Yes	602 (6.76)	598 (99.33)	4 (0.66)
**PAL **(MET hours per week)	24–36.5	2385 (26.78)	1422 (59.62)	963 (40.37)
	36.6–44.9	4555 (51.15)	1479 (32.46)	3076 (67.53)
	≥ 45	1965 (22.06)	1501 (76.38)	464 (23.61)
**BMI (** kg/m^2^**)**	< 18.5	179 (2.01)	101 (56.42)	78 (43.57)
	18.6–25	2565 (28.80)	1553 (60.54)	1012 (39.45)
	25.1–30	3868 (43.43)	2023 (52.30)	1845 (47.69)
	30.1–34.9	1805 (20.26)	617 (34.18)	1188 (65.81)
	> 35	488 (5.48)	98 (20.08)	390 (79.91)
**Kidney disease**	Yes	1574 (17.67)	902 (57.30)	672 (42.69)
**Depression**	Yes	275 (3.08)	66 (24.00)	209 (76.00)
**Psychiatric disorder**	Yes	178 (1.99)	83 (46.62)	95 (53.37)
**CVD**	Yes	228 (2.56)	99 (43.42)	129 (56.57)

*PA*, physical activity level, *BMI* body mass index, *CVD* cardiovascular disease

**Table 2:** Minimum and maximum duration of mobile phone use and changes in systolic and diastolic blood pressure and heart rate in participants based on the duration of mobile phone use

Variables		Total	Min–Max^*^	Non-users	Q1	Q2	Q3	Q4	-*P* value for trend^**^
**Population size, n (%)**		8905 (100)	-	1515 (17.0)	1949 (21.8)	1733 (19.4)	1911 (21.4)	1797 (20.1)	-
**Mean minute per da (min- max)**		19.5 (0–330)	-	**-**	3.4 (0.5–6)	8.0 (6.5–10)	16.2 (10.5–22.5)	50.4 (23.5–330)	-
**SBP (mmHg) (Mean ± SD)**	**Male**	107.9 ± 15.5	101.2–139.9	113.3 ± 16.7	109.1 ± 16.1	107.8 ± 15.0	104.3 ± 11.3	101.5 ± 13.2	0.065
	**Female**	104.2 ± 15.7	94.2–139.8	106.6 ± 17.3	105.5 ± 15.0	100.5 ± 14.8	96.2 ± 14.4	89.7 ± 12.5	** < 0.001**
**DBP (mmHg) (Mean ± SD)**	**Male**	70.0. ± 9.9	68.9–89.4	73.5 ± 10.4	70.5 ± 9.7	68.7 ± 9.8	66.5 ± 10.0	64.8 ± 9.9	0.886
	**Female**	67.8 ± 9.4	61.2–89.6	69.2 ± 10.1	68.2 ± 9.2	64.3 ± 9.1	61.1 ± 8.7	57.9 ± 9.1	** < 0.001**
**Heart rate (BPM) (Mean ± SD)**	**Male**	72.2 ± 9.1	67.2–78.4	71.6 ± 8.8	73.2 ± 6.0	73.9 ± 9.3	74.1 ± 9.0	74.6 ± 7.0	0.955
	**Female**	75.2 ± 9.8	73.3–89.6	74.9 ± 9.9	76.8 ± 8.0	75.9 ± 9.3	73.3 ± 9.7	71.8 ± 9.7	**0.045**

*Q* Quartile, *SBP* Systolic blood pressure, *DBP* Diastolic blood pressure, *mmHg* Millimeters of mercury, *BPM* Beat per minute

^*^ Minimum and Maximum of blood pressure

^**^ *P*-value was obtained by the Chi-square test for trend and *P* < 0.05 were considered as statistically significant

### Association between mobile phone use duration and blood pressure and heart rate

**Fig. 1:**
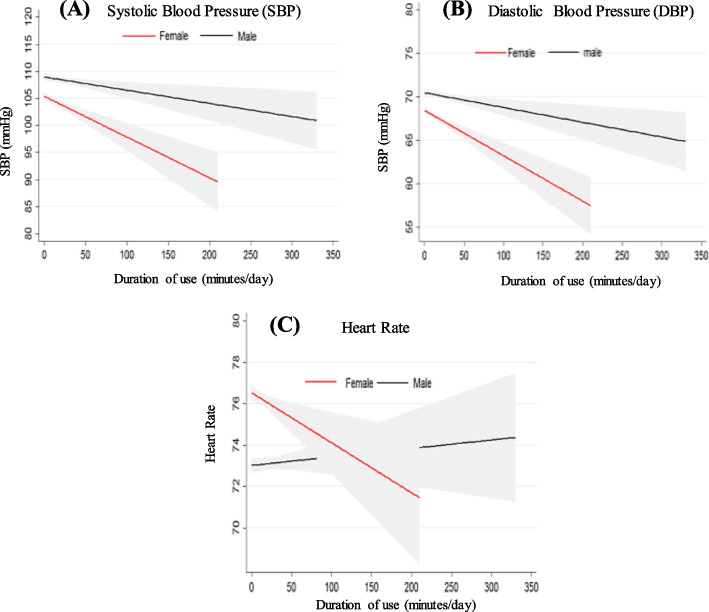
The dose–response relationship between duration of mobile phone use and systolic blood pressure (A), diastolic blood pressure (B), and heart rate (C)

Our findings revealed a dose–response relationship between mobile phone use time and DBP, SBP, and HR in females. Also, assessment of the linear relationship between duration of mobile phone use and blood pressure and HR showed a significant decreasing trend in the mean SBP and DBP (*p* < 0.001) and HR (*p* = 0.045) in women with an increase in the duration of mobile phone use. As shown in Table 2 and Fig. 1, SBP and DBP were significantly lower in women who used the mobile phone longer than those who did not use it. Similarly, the HR was significantly reduced by an increase in mobile phone use time in females. Also, a decreasing trend in the studied parameters was observed in men, but these values were not statistically significant. Although the average mobile phone use time in males was higher than in females, the reduction of SBP and DBP in females was significantly more than in males. Besides, the variation of HR numbers was higher than SBP and DBP (Table 2).

### Multivariate analysis for mobile phone use duration and blood pressure and heart rate

In order to observe the net effect of the duration of mobile phone usage on the occurrence of blood pressure, some confounding factors were adjusted. After adjustment for main confounding factors in model 1 (adjusted for age, gender, smoking status, residential areas, education level, and socioeconomic status), model 2 (adjusted for variables in model 1 plus BMI, and physical activity), and model 3 (adjusted for variables in model 2 plus kidney disease, depression, psychiatric disorder, and CVD), SBP [-2.52 (-4.11, -0.94)] and DBP [-1.86 (-2.83, -0.89)] in individuals who used their mobile phone more than 8 h/day (Q2-Q4) in comparison to those who never used (Crude) were significantly decreased in model 1, 2 and 3. However, the results of all three models for heart rate didn’t show a significant association between mobile phone usage duration and heart rate [-0.89 (-1.93, 0.15)] (Table 3).

**Table 3:** Findings from the linear regression for the association of mobile phone usage time and systolic, diastolic blood pressure, and heart rate

**Variables**		**Participants (n)**	**Crude** ^a^ **β (CI 95%)** ^e^	**Model 1** ^b^ **β (CI 95%)**	**Model 2** ^c^ **β (CI 95%)**	**Model 3** ^d^ **β (CI 95%)**
**Systolic blood pressure**	**Non-users** ^d^	1515	1	1	1	1
	**Q1**	1949	-2.73 (-4.05, -1.42)	-0.47 (-1.79, 0.86)	-0.75 (-2.05, 0.55)	-0.82 (-2.12, 0.49)
	**Q2**	1733	-3.06 (-4.41, -1.7)	-0.98 (-2.35, 0.39)	**-1.49 (-2.84, -0.14)**	**-1.64 (-3.00, -0.28)**
	**Q3**	1911	-3.4 (-4.74, -2.05)	-0.73 (-2.12, 0.66)	**-1.16 (-2.53, -0.21)**	**-1.17 (-2.55, -0.19)**
	**Q4**	1797	-5.14 (-6.68, -3.59)	**-2.05 (-3.65, -0.46)**	**-2.45 (-4.03, -0.87)**	**-2.52 (-4.11, -0.94)**
**Dystopic blood pressure**	**Non-users**	1515	1	1	1	1
	**Q1**	1949	-1.22 (-2, -0.43)	-0.04 (-0.84, 0.77)	-0.13 (-0.93, 0.67)	-0.13 (-0.93, 0.67)
	**Q2**	1733	-1.93 (-2.74, -1.11)	-0.8 (-1.64, 0.03)	**-1.03 (-1.86, -0.2)**	**-1.07 (-1.9, -0.24)**
	**Q3**	1911	-2.16 (-2.96, -1.35)	-0.68 (-1.53, 0.16)	**-0.88 (-1.72, -0.04)**	**-0.85 (-1.7, -0.01)**
	**Q4**	1797	-3.36 (-4.28, -2.43)	**-1.66 (-2.63, -0.69)**	**-1.82 (-2.79, -0.86)**	**-1.86 (-2.83, -0.89)**
**Heart rate**	**Non-users**	1515	1	1	1	1
	**Q1**	1949	1.29 (0.47, 2.11)	0.82 (-0.03, 1.67)	0.83 (-0.03, 1.68)	0.8 (-0.06, 1.65)
	**Q2**	1733	0.08 (-0.77, 0.93)	-0.32 (-1.21, 0.56)	-0.39 (-1.28, 0.49)	-0.47 (-1.36, 0.42)
	**Q3**	1911	0.4 (-0.44, 1.24)	0.03 (-0.87, 0.93)	0 (-0.9, 0.9)	-0.06 (-0.96, 0.84)
	**Q4**	1797	-0.48 (-1.44, 0.49)	-0.87 (-1.89, 0.16)	-0.87 (-1.9, 0.17)	-0.89 (-1.93, 0.15)

*CI* confidence interval, *Q* Quartile

The linear regression test was used for analysis

^a^ Unadjusted

^b^ **Model 1**. Adjusted for age, gender, smoking status, residential areas, education level, socioeconomic status

^c^ **Model 2**. Adjusted for variables in model 1 plus BMI and physical activity

^d^ **Model 3**. Adjusted for variables in model 2 plus kidney disease, depression, psychiatric disorder and CVD

^e^ 95% Confidence Intervals

^d^ as reference quartile

## Discussion

In the present study, a significant decreasing trend in the SBP, DBP, and HR by increasing the duration of mobile phone use in females was found. In the same way, a decrease in the studied parameters was observed in men, but these values were not statistically significant. According to these results, people who use mobile phones for a longer period of time may have lower blood pressure and HR than non-users. There are several studies in this field that are consistent with our results [7, 18, 26, 27]. For instance, a cross-sectional study by Stalin et al. investigated the relationship between mobile phone use and health problems such as hypertension, headache, and earache in 2121 participants. Their findings showed a negative and significant relationship between mobile phone use and blood pressure with a 25% reduction in the risk of hypertension as a result of using mobile phones [18]. In another cross-sectional study by Suresh et al. that was conducted on 21,135 adults, an inverse association was reported between mobile phone use and hypertension independent of age, sex, race/ethnicity, smoking, alcohol consumption, education, BMI, and physical activity. This relationship was more significant in the women aged below 60 years with a BMI below 25 kg/m^2^ [7]. Increasing parasympathetic activity and decreasing sympathetic activity in the brain stem along with improving social relationships are the possible mechanisms leading to a decrease in blood pressure with increasing time spent using mobile phones. Besides, mobile phone users communicate regularly with other people and have an active relationship with the community. This active connection can have protective effects on CVD and health by reducing their stress level [28]. However, this observed inverse relationship between mobile phone use and blood pressure may be accidental in nature and needs more comprehensive survey [7, 28, 29]. Furthermore, according to an investigation by Crippa et al., when someone calls on a mobile phone, SBP increases significantly from 121.77 to 129.82, and for reasons that have not yet been determined, the amount of SBP was lower in users who were accustomed to more than 30 phone calls a day. They suggested two possible reasons for this, the first possibility was that most people using mobile phones are young and less exposed to high blood pressure and the adverse effects of mobile phones on the cardiovascular system, and the second reason presented by them was that people who make more than 30 calls per day may feel more assured about not missing opportunities by constantly using their mobile phones [30].

In addition, electromagnetic fields alter the electrical activity of the brain and heart. Mobile phones have been shown to emit a type of non-ionizing electromagnetic radiation that can be absorbed by body tissues and increase brain glucose metabolism. Accordingly, different in vivo studies showed a significant increase in SBP, DBP, mean arterial blood pressure, total cholesterol, atherogenic indices, and heart NO levels after exposure to electromagnetic field radiation from a dual transceiver mobile phone (DTrMP) and also 2.45 GHz of wireless fidelity (WIFI) radiations [31, 32]. The possible mechanism for this increasing effect may be attributed to an increase in serum lipid profiles that can lead to an increase in atherogenic indices, peripheral resistance, or heart muscle sympathetic activity. Moreover, electromagnetic exposure may affect heart rate and blood pressure via direct and indirect mechanisms. The direct mechanism is related to effect of electromagnetic field on divalent mineral flux such as Ca^++^ and Zn^++^ homeostasis. Exposure to electromagnetic field accelerate Ca^++^/calmodulin-dependent myosin light chain phosphorylation. Through indirect mechanism, RF modulates the autonomic nervous system, plasma catecholamines, and glucocorticoids [32–34].

The amount of energy that mobile phone users are exposed to depends on factors such as the type of mobile technology, the distance between the phone and the user, the amount and type of mobile phone use, and the user's distance from the mobile towers [35, 36].

Nevertheless, some studies have shown that mobile phone use and exposure to electromagnetic radiation may have no adverse effect on users' blood pressure, HR, and cardiovascular system. In this way, Tahvanainen et al. in a randomized, double-blind trial, evaluated the blood pressure and HR of 32 healthy people after 35 min’ exposure to 900 MHz (2 W) and 1800 MHz (1 W) cellular phone irradiation in comparison to sham exposure in separate sessions. They indicated that arterial blood pressure and HR didn’t change significantly during or after the 35 min radiofrequency exposures at 900 MHz or 1800 MHz, compared to sham exposure [26]. In another study, Magiera and Solecka by investigating the effects of electromagnetic radiation on human health suggested that there is no proven evidence about the negative effects of electromagnetic fields on brain activity, sleep, HR, cognitive function, and blood pressure [27]. On the other hand, our findings may be conflicting with different previous studies in this area and our knowledge of the subject [8, 9]. In this regard, some studies examined the mobile phone, smartphone, and internet use on blood pressure in adolescence [37–40]. The findings of these studies showed a significant and positive association between mobile phone use with headache, earache, tinnitus, and restlessness. Their findings revealed that excessive use of mobile phones may increase tachycardia by 28% and blood pressure by 23%. Also, anemia, thrombocytopenia, and increased red cell distribution width (RDW) are other problems in people who use cell phones excessively [18, 41]. A possible explanation for this effect is that time spent on mobile phones takes time away from other activities and health-related behaviors, such as physical activity, positive interpersonal relationships, or staying focused at work or school. It is equally possible that excessive mobile phone use may negatively affect people by causing stress, anxiety, insomnia (reduced sleep duration and quality), sedentary time, changes in posture and pain, and reduced mental wellbeing, which may lead to cardiovascular problems and elevated blood pressure [42–46]. In the same way, Ekici et al. examined the effect of mobile phone use duration on heart rate variability (HRV) in 148 healthy individuals that used mobile phones for more than 10 years. All individuals were divided into four groups based on mobile phone duration use including the control group (no mobile phone use), Group 1 (< 30 min/day), Group 2 (30–60 min/day), and Group 3 (> 60 min/day). They demonstrated that the duration of mobile phone use may affect the autonomic balance in healthy subjects and the electromagnetic field created by mobile phones may induce HRV changes in the long term [47]. Likewise, the results of a case–control study about the relationship between mobile phone use and blood pressure in Indian adults showed that the duration of mobile phone use and the number of calls per day were significantly associated with the risk of developing hypertension. Also, the risk of developing high blood pressure in people who have used their phone for at least eight years and whose calls have lasted more than 60 min/day increases by 6 and 4 times, respectively [5]. Another study by Giuseppe et al. reported an increase in SBP and DBP from 121.77 to 121.82 mmHg in 94 patients with mild-to-moderate hypertension after answering their mobile phones. They suggested that talking on mobile phones can significantly increase blood pressure [48]. Moreover, Zou et al. studied the association between hypertension and smartphone addiction among 2639 junior school students. They reported that smartphone addiction is significantly and independently associated with hypertension and suggested that smartphone addiction may be a new risk factor for high blood pressure in adolescence [39]. According to different studies, there are various mechanisms besides electromagnetic waves in the occurrence of blood pressure and heart rate due to the use of mobile phones, each of them affects the nervous and cardiovascular systems separately. Since the effects of physical activity, higher levels of anxiety, and other factors associated with stimulation of the sympathetic nervous system in the development of hypertension and increased heart rate have been identified, in this study we tried to investigate the unknown effects of mobile phone use in the development of hypertension. The present study showed a significant decrease in HR with increasing mobile phone use in women. Similarly, other studies reported the inverse association between mobile phone use and exposure to 60-Hz electromagnetic fields with HRV parameters in healthy subjects [41, 47, 49, 50].

Opposing the results of the above studies, our findings showed that the mobile phone use duration has a negative association with blood pressure. Since other important factors such as nutrition, underlying diseases, genetics, and psychological factors can affect blood pressure, so one of the reasons for the difference between the results of our study and other investigations may be due to differences in the type of nutrition, lifestyle change or other predisposing factors in the people of the study area. Therefore, by conducting and comparing the results of comprehensive studies in different geographical areas with diverse populations, and by considering all the factors affecting the incidence of hypertension, we can better explain the effect of mobile phone use duration on blood pressure. Another reason for the different results in our study compared to other studies may be due to the small sample size in these studies and the method used, as well as the difference between the mobile phone models used in these studies.

In recent years, the use of smartphones has increased significantly in different societies, including Iran, so the effects of using smartphones on blood pressure may be different from ordinary mobile phones. It seems that smartphone use may affect blood pressure levels via reduced physical activity, increased anxiety levels, and forward head posture [46]. Other studies showed that excessive smartphone use alters brain activity by changing functional connectivity between regions related to cognitive control and reward prediction. Also, by using fMRI, a negative relationship was observed between orbitofrontal cortex (OFC) connectivity with the nucleus accumbens (NAcc) and internet use withdrawal symptoms and cortisol concentrations. Moreover, a positive correlation was reported between the node centrality of the right amygdala and smartphone dependence as well as sleep problems and depression symptoms [51–53]. Since we did not have access to people's mobile phone models, it was not possible to compare the effects of different mobile phones in this study. Additionally, with the development of wireless communications, the use of smartphones by people is increasing worldwide. Because these phones use 4G and 5G network technology for a faster communication, comprehensive studies are needed to examine the effects of this advanced technology on people's health. In this regard, in three U.S states and many European countries, specific policies and guidelines have been developed for the use of cell phones and other wireless devices in order to increase the health of users [54]. Unfortunately, there are no specific policies or guidelines for the use of mobile phones in some developing countries, and it is hoped that the results of this study and similar more comprehensive studies on smartphones will lead to the development of new laws and policies in these countries.

## Strengths and limitations

The primary strength of this study was the large sample size for both sexes that evaluated the data from the cohort study. Also, the present population-based study pioneered the investigation of the relationship between mobile phone use and blood pressure in Iran. However, there were some limitations regarding the methodology and nature of the present study. The primary limitation of this study was failing to access information about other sources of electromagnetic waves, including satellite waves, microwaves, radio-frequency waves, and television waves, as potential confounding variables. Besides, there was a lack of data about the models of mobile phones (cell phones or smartphones) and specific absorption ratios of mobile phones. As well, nutritional factors that could play an important role in participants' blood pressure were ignored. Another limitation of this study was the difference between called and answered calls which may affect the results of the study. Using the retrospective direct question method for data collection which is based on the subjects’ memory increases the possible misreporting and can be considered as another limitation of our study. By considering the nature of cross-sectional studies, in the present study, we were unable to infer causality between mobile phone use and blood pressure, and HR. Therefore, further studies are recommended to be conducted to confirm this relationship.

## Conclusion

The findings of the present study revealed that mobile phone use duration significantly affect BP just in females. After adjustment for all confounding factors, in whole population, SBP and DBP, in individuals who used their mobile phone more than 8 h/day (Q2-Q4), were significantly decreased in all three models. In contrast, after adjustment for all confounding factors, there was no significant association between mobile phone use duration and HR in all adjusted models. Since the association between mobile phone use duration and HR was weak for women and absent for men, perhaps this was just a chance finding that needs to be investigated further.

## Data Availability

The datasets used and/or analyzed during the current study are available from the corresponding author on reasonable request.

## Abbreviations

BMI: Body mass index
CI: Confidence Intervals
CVD: Cardiovascular disease
DBP: Diastolic Blood Pressure
DTrMP: Dual transceiver mobile phone
HR: Heart rate
HRV: Heart Rate Variability
ICT: Information Communication Technology tool
METs: Metabolic equivalents
NAcc: Nucleus accumbens
NHIS: National Health Interview Survey
OFC: Orbitofrontal Cortex
PAL: Physical Activity Level
PCA: Principal Component Analysis
Q: Quartile
RaNCD: Ravansar Non-Communicable Diseases
RDW: Red cell distribution width
SBP: Systolic Blood Pressure
WIFI: Wireless fidelity
